# Buyang Huanwu decoction facilitates neurorehabilitation through an improvement of synaptic plasticity in cerebral ischemic rats

**DOI:** 10.1186/s12906-017-1680-9

**Published:** 2017-03-28

**Authors:** Ruihuan Pan, Jun Cai, Lechang Zhan, Youhua Guo, Run-Yue Huang, Xiong Li, Mingchao Zhou, Dandan Xu, Jie Zhan, Hongxia Chen

**Affiliations:** 1 Department of Rehabilitation, Hospital of Guangzhou Higher Education Mega Center, Guangdong Provincial Hospital of Chinese Medicine, No. 55 Neihuan Xi Road, Guangzhou, Guangdong 510006 China; 2 Department of Neurosurgery, Hospital of Guangzhou Higher Education Mega Center, Guangdong Provincial Hospital of Chinese Medicine, No. 55 Neihuan Xi Road, Guangzhou, Guangdong 510006 China; 3 Department of Rheumatism, Hospital of Guangzhou Higher Education Mega Center, Guangdong Provincial Hospital of Chinese Medicine, Guangzhou, 510006 China; 4 Phase 1 Clinical Research Center, Guangdong Provincial Hospital of Chinese Medicine, Guangzhou, 510120 China; 5 The Second Institute of Clinical Medicine, Guangzhou University of Chinese Medicine, Guangzhou, 510120 China; 6 Post-doctoral Research Center of Guangzhou University of Chinese Medicine, Guangzhou, 510006 China

**Keywords:** Buyang Huanwu decoction, Cerebral ischemia, Neurorehabilitation, Synaptic plasticity

## Abstract

**Background:**

Loss of neural function is a critical but unsolved issue after cerebral ischemia insult. Neuronal plasticity and remodeling are crucial for recovery of neural functions after brain injury. Buyang Huanwu decoction, which is a classic formula in traditional Chinese medicine, can positively alter synaptic plasticity. This study assessed the effects of Buyang Huanwu decoction in combination with physical exercise on neuronal plasticity in cerebral ischemic rats.

**Methods:**

Cerebral ischemic rats were administered Buyang Huanwu decoction and participated in physical exercise after the induction of a permanent middle cerebral artery occlusion. The neurobehavioral functions and infarct volumes were evaluated. The presynaptic (SYN), postsynaptic (GAP-43) and cytoskeletal (MAP-2) proteins in the coronal brain samples were evaluated by immunohistochemistry and western blot analyses. The ultrastructure of the neuronal synaptic junctions in the same region were analyzed using transmission electron microscopy.

**Results:**

Combination treatment of Buyang Huanwu decoction and physical exercise ameliorated the neurobehavioral deficits (*p* < 0.05), significantly enhanced the expression levels of SYN, GAP-43 and MAP-2 (*p* < 0.05), and maintained the synaptic ultrastructure.

**Conclusions:**

Buyang Huanwu decoction facilitated neurorehabilitation following a cerebral ischemia insult through an improvement in synaptic plasticity.

**Graphical abstract:**
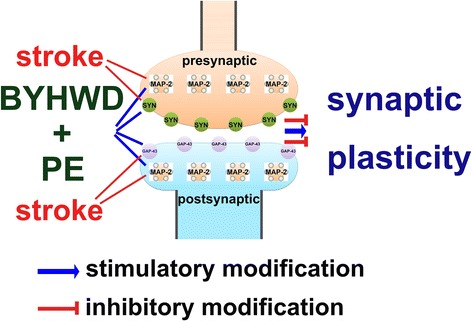
The Buyang Huanwu decoction (*BYHWD*) combined with physical exercise (*PE*) attenuates synaptic disruption and promotes synaptic plasticity following cerebral ischemia (*stroke*).

**Electronic supplementary material:**

The online version of this article (doi:10.1186/s12906-017-1680-9) contains supplementary material, which is available to authorized users.

## Background

Cerebral ischemia is a major cause of adult mortality and morbidity in developed countries [1–3]. Although numerous drugs and therapies have been tested, there is no effective treatment that dramatically attenuates the neural deficits after stroke in the clinical setting. Because the central nervous system (CNS) of adult mammals has limited self-repair or regeneration, a CNS injury inevitably results in a loss of neural function [4, 5]. Neuronal plasticity and remodeling are critical processes that underlie normal CNS function [6]. The CNS can facilitate the neuronal plasticity induced by brain injury [7]. The decreased synaptic activity after brain injury leads to increased presynaptic release of neurotransmitters, as well as upregulated postsynaptic response to those neurotransmitters; this can, at least in part, restore neural function following an injury [8]. In addition to synaptic activity regulation, new synapse formation, which may compensate for the lost structural circuits, can be triggered by stroke [9–12]. This self-regulation of synaptic plasticity is activity- and experience-dependent [7]. Physical exercise improves the neurological impairments that follow brain insults [13–15]. In synaptic mechanism studies, the beneficial effects of physical exercise were correlated with the maintenance of pre- and postsynaptic components [16, 17].

Pharmacological compounds can assist the recovery from stroke and other CNS diseases [18–20]. Numerous pharmaceutical drugs have been studied, but few have dramatically positive results. The Buyang Huanwu decoction (BYHWD) has been utilized for hundreds of years to improve the recovery of neurological function in stroke-induced disabilities in China [21]. This formulation consists of: Radix Astragali, the root of *Astragalus membranaceus* (Fisch.) Bge. var. *mongholicus* (Bge.) Hsiao; Radix Angelicae Sinensis, the root of *Angelica sinensis* (Oliv.) Diels*;* Radix Paeoniae Rubra*,* the root of *Paeonia lactiflora* Pall.; Chuanxiong Rhizoma, the root and rhizome of *Ligusticum chuanxiong* Hort.; Semen Persicae, the seed of *Prunus persica* (L) Batsch; Flos Carthami, the flower of *Carthamus tinctorius* L. and Pheretima, the dried body of *Pheretima aspergillum* (E. Perrier) [22, 23], The protective mechanisms underlying this classic traditional Chinese formula includes the stimulation of neural proliferation, the modulation of VEGF and Flk1 expression levels, alterations in glutamate levels, and a selective decrease of some amino acids in the cerebrospinal fluid [4, 22, 24]. BYHWD was also testified to be neuroprotective in the cerebral ischemic models in many other studies. A study reported oral administration of BYHWD inhibits caspase-3 and neuron apoptosis in the hippocampal CA1 region of Wistar rats following four-vessel occlusion (4-VO) [25]. Intragastric administration of BYHWD down-regulates inflammation, apoptosis and angiogenesis, as well as up-regulates neurogenesis and nervous system development in cerebral ischemia/reperfusion (CI/R) injured mice and rats [21, 22]. Despite its clinical and laboratory effectiveness, the functional recovery using BYHWD following cerebral ischemia has not been fully investigated. It is difficult to draw a definite conclusion on safety of BYHWD, admittedly, BYHWD seems well tolerable to all patients in clinically [26]. In addition, the safety of BYHWD on patients with acute cerebral ischemia has been testified in a Chinese clinical trial [27].

In this study, we evaluated the combination of BYHWD and physical exercise in cerebral ischemic rats, which had a permanent middle cerebral artery occlusion. We assessed the therapeutic effects of this combination therapy by measuring neurobehavioral functions and infarct volumes, analyzing the SYN, GAP-43 and MAP-2 expression levels, and evaluating the synaptic ultrastructure.

## Methods

### Animals

The animal handling was approved by the Institutional Animal Care and Use Committee (IACUC) of the Guangdong Provincial Hospital of Chinese Medicine, Guangzhou, China. This experimental protocol is in agreement with the European Community guidelines (EEC Directive of 1986; 86/609/EEC). Adult male Sprague-Dawley rats were provided by the Medical Laboratory Animal Centre of Guangdong (Guangzhou, China). The animals (250–300 g) were housed in an animal room (12:12 h light/dark circle; 22–24 °C) with free access to food and water.

### Induction of the permanent middle cerebral artery occlusion (MCAO)

**Fig. 1:**
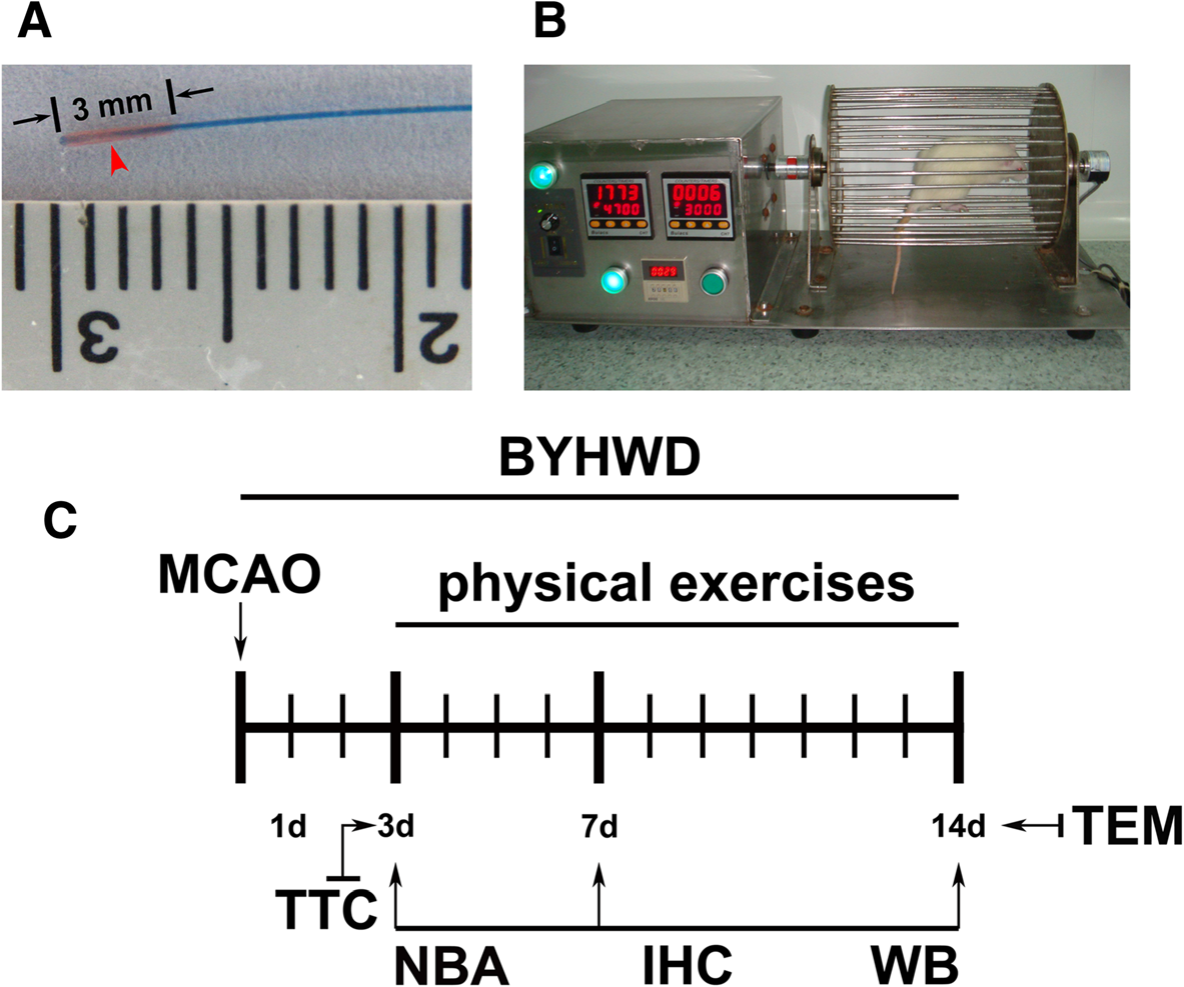
Laboratory techniques used in this study. A 4–0 suture coated with silicone rubber (**a**) was used for the MCAO induction. A programmable and motorized running wheel apparatus (**b**) was employed for the physical exercises post-cerebral ischemia. The design of this study (**c**). The intragastric administration of BYHWD began 2 h after the MCAO induction and was administered daily from Day 1 to 14 or until euthanasia. The physical exercises began on Day 3 post-ischemia and ended on Day 14 or euthanasia. The analytic modalities (neurobehavioral assessment, immunohistochemistry, and western blot) were performed on Day 3, 7 and 14 after the MCAO induction. Other techniques (TTC and TEM) were performed on Day 3 or 14 after the MCAO induction. BYHWD, Buyang Huanwu decoction; IHC, immunohistochemistry; MCAO, middle cerebral artery occlusion; NBA, neurobehavioral assessment; TEM, transmission electron microscope; TTC, triphenyltetrazolium chloride staining; WB, western-blot

The animals were anesthetized with ketamine (100 mg/kg; Gu-Tian Pharmaceutical Co., Ltd.; Fujian, China) and xylazine (10 mg/kg; Sigma-Aldrich; St. Louis, MI, USA). The animals’ temperature was maintained at 37.0 ± 0.5 °C using a heating pad (RWD Life Science; Shenzhen, China). The animals were placed in a supine position after anesthesia. Subsequently, under an operating microscope (Carl-Zeiss; Jena, Germany), a midline incision was made in the neck of the experimental subject. The right common carotid artery (CCA), external carotid artery (ECA) and internal carotid artery (ICA) were isolated. The ECA was cut approximately 5 mm above the common carotid artery bifurcation. We inserted a 4–0 suture (20 mm; Dermalon, 1744–31; Covidien; OH, USA) coated with silicone rubber (3 mm length, 0.4 mm diameter; Heraeus Kulzer; Hanau, Germany) into the ECA stump, where it was reversed into the ICA and finally to the ostium of the middle cerebral artery (MCA) (approximately 15 mm from the carotid bifurcation), as reported previously [28–30]. The 4–0 suture coated with silicone rubber is displayed in Fig. 1a.

### The laser Doppler flowmetry (LDF) analysis for the measurement of the surface cerebral blood flow (CBF)

A successful occlusion was confirmed by monitoring the cerebral blood flow (CBF) in the ipsilateral cortex. The measurement of the surface CBF was conducted by a laser Doppler flowmetry (LDF) (Moor Instruments; Axminster, UK) during the MCAO induction, as previously described [31, 32]. In order to determine the surface CBF that was supplied by the bilateral MCA, 2 burr holes (ID =2 mm) were drilled 2.5 mm to the left and right, and 1.0 mm posterior to bregma, as previously described [31, 32]. When the value of the ipsilateral CBF decreased to 1/3 of the baseline value, the MCA was considered to be successfully occluded.

### Buyang Huanwu decoction (BYHWD) preparation

The ingredients of BYHWD including Radix Astragali, Radix Angelicae Sinensis, Radix Paeoniae Rubra, Chuanxiong Rhizoma, Semen Persicae, Flos Carthami and Pheretima were mixed together at a ratio of 120:10:10:10:10:10:4.5 (dry weight). It was prepared as previously reported [4, 21]. All dried, crude drugs were ordered from Kang Mei Pharmaceutical Co., Ltd. (Puning, Guangdong, China) and were identified by the Department of Pharmacology at Guangdong Provincial Hospital of Chinese Medicine. To maintain the consistency of the herbal chemical ingredients, all herbs in this decoction were obtained from their original sources. The extraction procedure was performed according to the Chinese Pharmacopoeia. The details of the BYHWD ingredients were listed in Table 1, while the chemical fingerprints of the BYHWD ingredients were shown in the Additional file 1. The BYHWD was made by boiling the dried ingredients in distilled water at 100 °C for 30 min. A freeze-dried drug solution was subsequently made, and the BYHWD powder was produced under vacuum. The powder was dissolved in distilled water at a final concentration of 2.0 g/ml, which is equivalent to the dry weight of the raw materials.

**Table 1:** Ingredients of BYHWD

Components (Latin name)	Family	Voucher specimen No.	Part used	processing
**Plants**				
Radix Astragali	Leguminosae	130,401,831	Root	Extraction
Radix Angelicae Sinensis	Umbelliferae	130,504,671	Root	Extraction
Radix Angelicae Sinensis	Ranunculaceae	130,409,121	Root	Extraction
Radix Paeoniae Rubra	Umbelliferae	130,509,661	Root and rhizome	Extraction
Semen Persicae	Rosaceae	130,308,751	Seed	Extraction
Flos Carthami	Composite	130,314,541	Flower	Extraction
**Insects**				
Pheretima	Megascolecidae	130,608,701	Dried body	Farina

### Physical exercise

To quantify the physical exercise intensity, a programmable and motorized wheel apparatus (21 cm in diameter, 40 cm in length; Fig. 1b) was used. For pre-conditioning, the animals used the running wheel for exercise for 7 days prior to the MCAO induction. On the first day, the rats ran in the wheel at a speed of 5 revolutions (rev)/min (approximately 3 m/min). The speed of the wheel gradually increased to 10 rev/min (approximately 6 m/min) on Day 7, as reported previously [13, 33].

### Animal groups and study design

The rats were randomly divided into 5 groups: 1) the sham group (*n* = 24), 2) the MCAO group (*n* = 38), 3) the MCAO + BYHWD group (*n* = 35), 4) the MCAO + physical exercise (PE) group (*n* = 36), and 5) the MCAO + BYHWD + PE group (*n* = 29). Before MCAO induction, the rats from the MCAO + PE and MCAO+ BYHWD + PE groups participated in physical exercise with the running wheel, as described above. The animals from the MCAO, MCAO + BYHWD, MCAO + PE and MCAO + BYHWD + PE groups underwent the permanent MCAO procedure. The sham group animals were given a sham surgery, which was identical to the MCAO induction procedure, without inserting the 4–0 silicone-coated nylon monofilament. The rats from the MCAO + BYHWD and MCAO + BYHWD + PE groups were administered BYHWD (20 g/kg) by intragastric administration. The medication began 2 h after the MCAO induction and was administered daily from 1 to 14 days or until euthanasia. The physical exercises were performed daily from 3 to 14 days post-ischemia in the MCAO+ BYHWD + PE group. The pattern of physical exercises were identical to the previous studies [13, 33].

According to previous study [22], we chose 10–40 g/kg as the dosage of BYHWD in the preliminary study (Cai J, Pan R, Xu D, Wang B, Zhou M, Chen H. unpublished data). We found that the neuroprotective effects (neurobehavioral assessments) in dosage of 20–40 g/kg were better than those in dosage of 10 g/kg. Therefore, the dosage of 20 g/kg was chosen in present study. In addition, we did not found any obvious side-effects in dosage of 10–40 g/kg in preliminary study (Cai J, Pan R, Xu D, Wang B, Zhou M, Chen H. unpublished data).

A subset of rats from all groups (*n* = 6 at each time point) underwent a neurobehavioral assessment on Day 3, 7 and 14 post-ischemia, and were then euthanized by decapitation for triphenyltetrazolium chloride (TTC) staining and infarct volume calculations (*n* = 5 on Day 3), immunohistochemical assays (*n* = 4 on Days 3, 7 and 14), western blot analyses (*n* = 6 on Days 3, 7 and 14) and transmission electron microscopic (TEM) examinations (*n* = 5 at Day 14). The experimental design was shown in Fig. 1c.

### Neurobehavioral assessment

We adopted a scale of 0–18 (normal score, 0; maximal deficit score, 18) for the neurobehavioral measurement to assess the neurological deficits on Days 3, 7 and 14 after the MCAO induction [33–35]. The neurological severity score was a combination of motor, sensory, reflex and balance tests. The details of the neurobehavioral assessment were displayed in Additional file 2.

### Infarct volume quantification

After euthanasia, the brains were extracted. The brain tissue was sliced into 2 mm cross-sections using a rat brain matrix (RWD, Life Science). The slices were incubated in a 2% solution of 2,3,5-TTC (Sigma-Aldrich; St Louis, MO, USA) at 37 °C for 20 min. The sample sections were photographed and analyzed by an observer blind to the group assignment using Image J software (NIH Program; Bethesda, MD, USA). The infarct volumes were calculated according to previously described methods [36].

### Immunohistochemical assay

Animals were euthanized and transcardially perfused with saline and 4% paraformaldehyde. The brains were removed and stored in 4% paraformaldehyde for 24 h. The brain sections (3 μm) were fixed in 4% paraformaldehyde for 5 min, and then washed and blocked for 30 min in 10% (*w*/*v*) bovine serum albumin dissolved in 0.1 M phosphate buffer saline (PBS). The sections were incubated at 4 °C overnight with the primary antibodies against SYN (SYN-1; 1:200 dilution; Cell Signaling Technology; Beverly, MA, USA), GAP-43 (1:200 dilution; Abcam; Cambridge, MA, USA) and MAP-2 (1:200 dilution; Santa Cruz Biotechnology; Dallas, TEX, USA), followed by an incubation with the corresponding Alexa Fluor 568 secondary antibody (goat anti-rabbit; 1:200 dilution; Abcam) at room temperature for 1 h. After washing in PBS, the coverslips were mounted on glass slides with 4,6-diamidino-2-phenylindole (DAPI;1:1000 dilution; Sigma-Aldrich).

### Western blot analysis

The frozen brain samples were lysed with a buffer containing 20 mM Tris (pH 7.6), 0.2% SDS, 1% TritonX-100, 1% deoxycholate, 1 mM phenylmethylsulfonyl fluoride, and 0.11 IU/mL aprotinin. All the ingredients were purchased from Sigma-Aldrich. The total protein was extracted from the brain samples and subjected to an 8% SDS-PAGE. The following antibodies were used: polyclonal antibodies against SYN (SYN-1; 1:1000 dilution; Cell Signalling Technology), GAP-43 (1:1000 dilution; Abcam), MAP-2 (1:1000 dilution; Santa Cruz Biotechnology) and β-actin (1:6000 dilution; Sigma-Aldrich). The densitometry analyses of the western blots were performed with Glyko Bandscan software (Glyko; Novato, CA, USA). The experiments were conducted at least 3 times.

### TEM examination

The animals were euthanized and transcardially perfused with 50 ml of saline followed by 100 ml of 4% paraformaldehyde and 2.5% glutaraldehyde in 0.1 mol/l cacodylic acid buffer (pH 7.3). Following an ethanol series, the fixed brain tissues (right frontal lobe) were dehydrated and embedded in an epoxy resin. Next, the samples were cut into ultrathin sections, which were subsequently mounted on copper grids and stained in uranyl acetate and citric acid lead. The prepared samples were imaged using a H-7650 transmission electron microscope (Hitachi; Tokyo, Japan).

### Statistical analyses

The data are shown as the means ± the standard error of the means (SEMs). A one-way analyses of variance (ANOVA) followed by a Student, Newman–Keuls or Dunnett’s post-hoc test, were utilized for the comparisons between more than 2 groups. SPSS 18.0 (SPSS; Chicago, IL, USA) was used for the statistical analyses, and the statistical significance was set at *p* < 0.05.

## Results

### Combination treatment of BYHWD and physical exercise decreased the mortality and ameliorated the neurological deficits

**Fig. 2:**
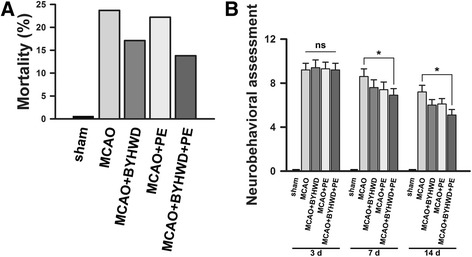
Comparisons of the mortalities and neurobehavioral assessment in all groups. Mortalities of all groups (**a**). Evaluations of the neurological deficits in all groups (*n* = 6 at each time point) (**b**). Scores of neurological deficits are expressed as the means ± standard error of the means; ns, not significant, *p* > 0.05; *, *p* < 0.05, compared to the MCAO group

Seventy-two hours after MCAO induction, the mortality rates varied among the 4 groups: 23.7% (9/38) in the MCAO group, 17.1% (6/35) in the MCAO + BYHWD group, 22.2% (8/36) in the MCAO + PE group, and 13.8% (4/29) in the MCAO + BYHWD + PE group (Fig. 2a). The mortality of the MCAO + BYHWD + PE group was lower than the other 3 groups.

On Days 3, 7, 14 post-ischemia, the neurological deficits of all groups were assessed using an 18-point system. A higher score indicated more deficits. We found that the MCAO group exhibited severe deficits (9.2 ± 0.6, 8.6 ± 0.7 and 7.2 ± 0.6 on Days 3, 7 and 14 post-ischemia, respectively). The MCAO + BYHWD group (9.4 ± 0.7, 7.6 ± 0.7 and 6.0 ± 0.5 on Days 3, 7 and 14 post-ischemia, respectively) and MCAO + PE group (9.3 ± 0.6, 7.4 ± 0.7 and 6.1 ± 0.5 on Days 3, 7 and 14 post-ischemia, respectively) exhibited moderate deficits. Noticeably, the MCAO + BYHWD + PE group exhibited milder neurological deficits at each time point relative to the other MCAO groups (9.2 ± 0.6, 6.9 ± 0.6 and 5.1 ± 0.5 on Days 3, 7 and 14 post-ischemia, respectively; Fig. 2b).

### Combination treatment of BYHWD and physical exercise did not reduce infarct volumes

**Fig. 3:**
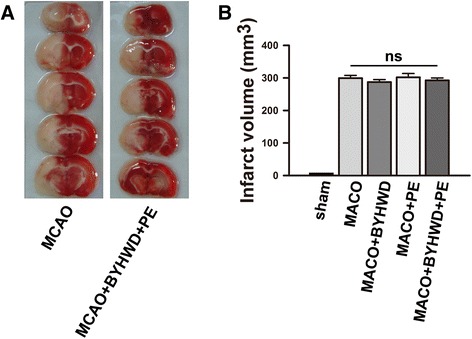
Representative TTC-stained slices and the infarct volumes in all groups. Representative TTC-stained slices of the coronal brain sections (**a**) from all groups exhibit the pale infarct area induced by a permanent MCAO. The infarct volumes in all groups on Day 3 after the MCAO induction (*n* = 5) (**b**). The bars indicate the means ± the standard errors of the means; ns, *p* > 0.05; *, *p* < 0.05, relative to the MCAO group

To determine whether combination treatment was neuroprotective, the infarct volumes of all groups were examined at each time point following the cerebral ischemia. The TTC-stained slices of the coronal brain sections were used to confirm the presence of successful cerebral infarctions in the MCAO induction animals. The representative examples were displayed in Fig. 3a. After TTC staining, the brain sections of sham group were uniformly stained a dark red and had no pale infarct area. On Day 3 post-ischemia, the infarct volumes in the MCAO, MCAO + BYHWD, MCAO + PE and MCAO + BYHWD + PE groups were 299.3 ± 8.5, 287.4 ± 7.6, 302.1 ± 11.5, 292.6 ± 7.2 mm3 respectively. There were no significant differences in the infarct volumes between these 4 groups (*p* > 0.05; Fig. 3b).

### Combination treatment increased SYN, GAP-43 and MAP-2 protein levels after cerebral ischemia

**Fig. 4:**
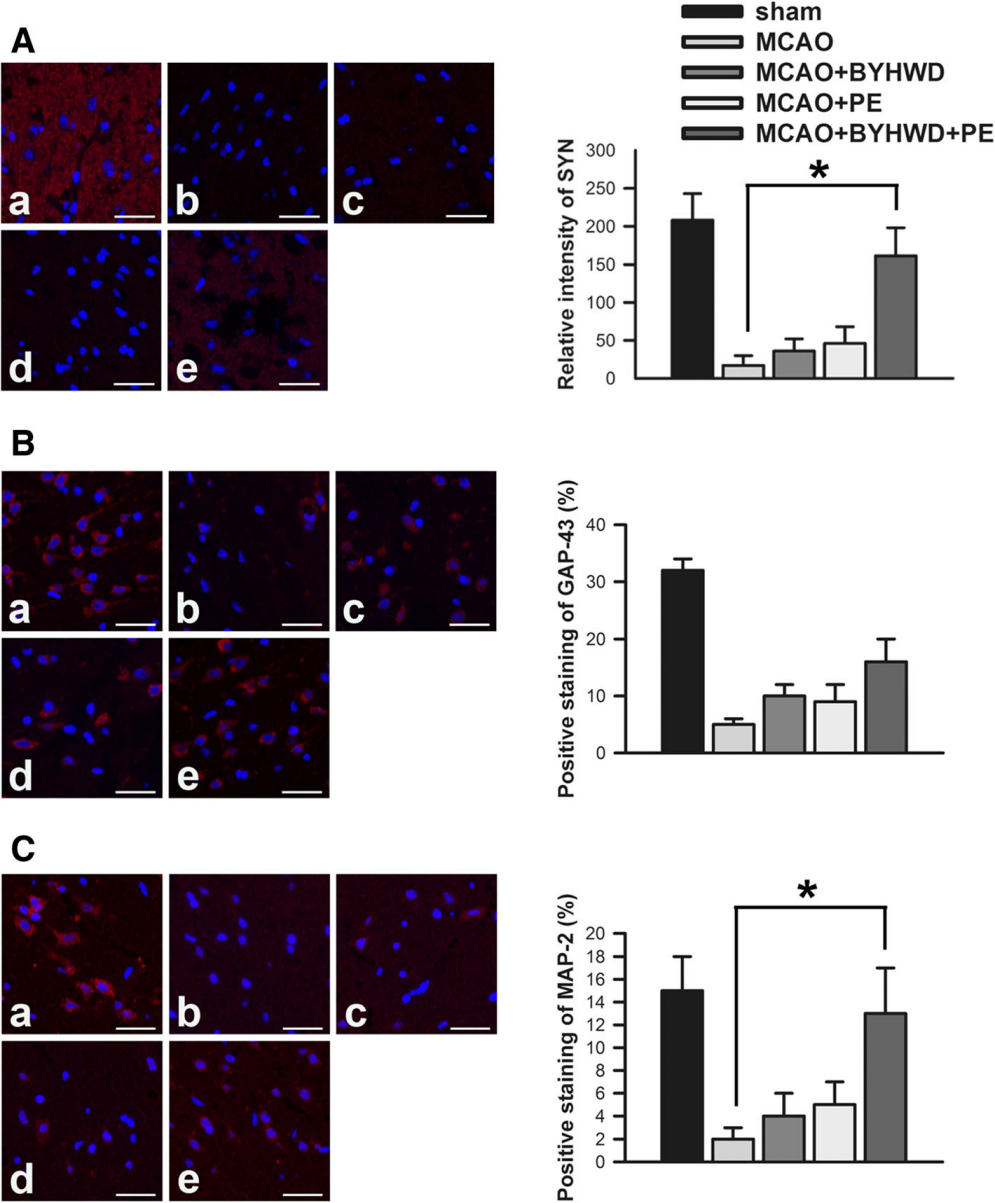
High magnification micrographs of the immunohistochemical fluorescent labeling of SYN (SYN-1), GAP-43 and MAP-2. The immunohistochemistry micrographs display the locations of the SYN (**a**), GAP-43 (**b**) and MAP-2 (**c**) proteins in sham (*a*), MCAO (*b*), MCAO + BYHWD (*c*), MCAO + PE (*d*) and MCAO + BYHWD + PE (*e*) groups. The scale bars =20 μm. The bars indicate the means ± the standard errors of the means; *, *p* < 0.05, relative to the MCAO group

Immunohistochemistry and western blots were employed to detect and quantify SYN (SYN-1), GAP-43 and MAP-2 protein levels. The immunohistochemical micrographs revealed that SYN, GAP-43 and MAP-2 proteins were normally expressed in the brain samples from sham group (Fig. 4A-a, B-a, C-a). However, in the micrographs of the peri-infarct zones from the MCAO group, the protein expression levels were dramatically reduced (Fig. 4A-b, B-b, C-b). The disruption of these proteins was mildly attenuated in the MCAO + BYHWD group (Fig. 4A-c, B-c, C-c) and the MCAO + PE group (Fig. 4A-d, B-d, C-d). The positive staining measurement of immunohistochemical micrographs were analyzed with Image J software (NIH Program, Bethesda, Maryland, USA). However, the positive staining measurement of SYN slices were employed relative intensity calculation technique as previously reported [37]. In the sections from the MCAO + BYHWD + PE group (Fig. 4A-e, B-e, C-e), the expression levels of these proteins were distinctly upregulated compared to the levels in the MCAO group (*p* < 0.05).

**Fig. 5:**
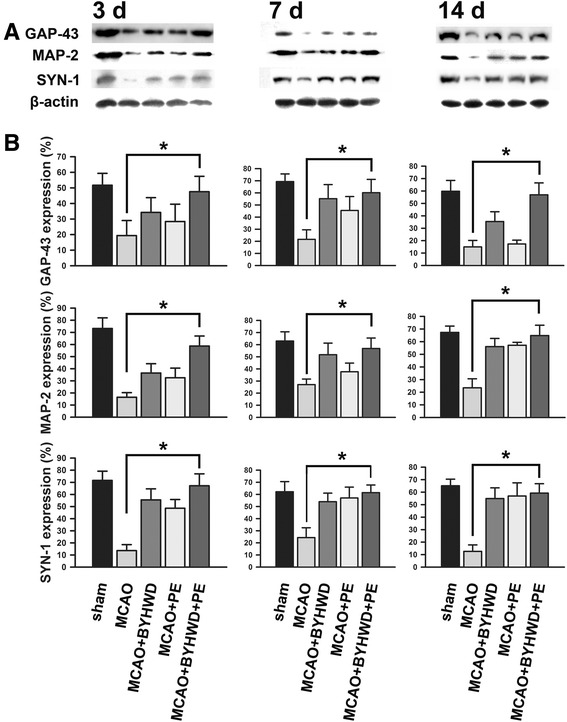
Expression levels of SYN (SYN-1), GAP-43 and MAP-2 in all groups at each time point. The proteins expression levels at each time point following the MCAO are presented in representative western-blotting autoradiograms (**a**). We detected SYN at 77 kDa, GAP-43 at 48 kDa, MAP-2 at 70 kDa and the β-actin loading control at 43 kDa. Lane 1, sham group; Lane 2, MCAO group; Lane 3, MCAO + BYHWD group; Lane 4, MCAO + PE group; Lane 5, MCAO + BYHWD + PE group. The quantitative analyses (*n* = 6) of the western blot results for the expression levels of SYN, GAP-43 and MAP-2 are presented in the bar graphs (**b**). The bars indicate the means ± standard errors of the means. *, *p* < 0.05, compared to the MCAO group

Western blot analyses revealed that the protein expression levels of SYN (SYN-1), GAP-43 and MAP-2 in the sham group were detectable. These protein expression levels were remarkably reduced in the MCAO group post-ischemia compared to the levels of the sham group (*p* < 0.05). However, the decreased levels of these proteins were ameliorated to a certain extent after the MCAO induction in the MCAO + BYHWD and MCAO + PE group, although not statistically significant (*p* > 0.05). In the MCAO + BYHWD + PE group, these proteins were distinctly up-regulated compared to the expression levels in the MCAO group (*p* < 0.05). The representative western blot micrographs were displayed in Fig. 5a, and the relative expression levels of SYN, GAP-43 and MAP-2 are disclosed in Fig. 5b.

### Combination treatment maintained the ultrastructural integrity of the synapse

**Fig. 6:**
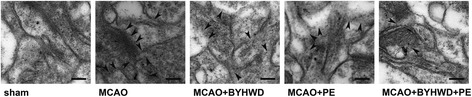
TEM micrograms. Representative synapse ultrastructures of all groups. An intact synaptic ultrastructure is shown in panel *sham*, and a disorderly synaptic ultrastructure is illustrated in panels *MCAO, MCAO + BYHWD*, *MCAO + PE* and *MCAO + BYHWD + PE*. The arrowheads presented in panels *MCAO*, *MCAO + BYHWD*, *MCAO + PE* and *MCAO + BYHWD + PE* indicate the synaptic disruptions. Scale bars =200 nm

TEM technique was used to analyze the ultrastructural changes of the synapse post-ischemia. The ultrastructure of the synapse in the sham group remained intact and clear. In the MCAO group, the axons and dendrites were chaotic, and there were no proper synapses in the peri-infarct region. In the MCAO + BYHWD and MCAO + PE groups, the axonal and dendritic membranes and the synaptic structures were healthier and more intact compared to the morphology found in the MCAO group. However, in the MCAO + BYHWD + PE group, the synaptic ultrastructure looked similar to the sham group’s ultrastructure, although there were some indistinct axons and dendritic membranes. The representative TEM micrographs were shown in Fig. 6.

## Discussion

The recovery after a CNS injury requires cortical plasticity, including synaptic plasticity [6, 7, 38]. Cerebral plasticity is influenced by many factors. Activity- and experience-dependent plasticity is very important for cerebral plasticity in both normal and injured CNS [10]. One treatment protocol that persistently induces a post-stroke behavioral recovery is the forced use of the disabled limbs in a patterned or task-specific behavioral activity task [7, 39–42]. This treatment strategy was developed using the theory of activity- and experience-dependent plasticity for the rehabilitation of the affected limbs. However, one treatment modality is unable to meet the extensive rehabilitative needs after stroke. Thus, pharmacological neuromodulation may lead to a significant improvement in rehabilitative outcomes [18, 43–45]. However, few drugs have been effective in facilitating physical rehabilitation. Buyang Huanwu decoction (BYHWD) is believed to be an important promoter in neurorehabilitation after stoke [27]. In this study, BYHWD was evaluated in synaptic plasticity.

According to Fig. 2a, the survival rates varied among the 4 MCAO groups. The mortalities of the 2 groups that were administered BYHWD were lower than the other MCAO groups. This finding agrees with a previous study [21]. However, the mortality rates of the MCAO mice reported by that study were approximately 50–83%, leading to a discrepancy in the total mortality between that study and the results presented here. This may be due to the anatomical variations of the posterior communicating artery in the MCAO mice, which can be optimized in future studies [46]. In this study, the physical exercise treatment was added after 3 days of cerebral ischemia. A neurobehavioral assessment scale was employed to evaluate the neurological deficits after MCAO induction [34, 35]. We found that the neurological deficits of the combination treatment group was similar to that of the other MCAO groups on Day 3 after the MCAO induction. It was remarkably alleviated on Day 7 and 14 post-ischemia, compared to the MCAO rats without treatment. This result indicates that BYHWD effectively facilitated the effects of the physical exercise after a cerebral ischemia insult.

SYN (SYN-1), GAP-43 and MAP-2 are associated with presynaptic and postsynaptic plasticity as well as the neuronal cytoskeleton [6, 38, 47, 48]. The synaptic plasticity markers SYN (SYN-1), GAP-43 and MAP-2 are up-regulated in the post-ischemic milieu, thus, they were postulated to play pivotal roles in neurorehabilitation [49–53]. We qualitatively analyzed these 3 proteins to determine the effects of the combination therapy of BYHWD and physical exercise on synaptic plasticity. The immunohistochemical data suggested that BYHWD or physical exercise alone can ameliorate the disruption of these 3 synaptic proteins that was induced by the MCAO induction. However, the effects of the individual treatments were not significant, while the combination treatment was. Furthermore, we quantitatively analyzed the changes of these 3 proteins with various treatment modalities using western blot. The combination of the BYHWD and physical exercise treatment alleviated the decreases in the SYN, GAP-43 and MAP-2 proteins after the cerebral ischemia. These changes were consistent with the neurological deficits in the various groups after the MCAO induction. It was reported that there were differences in synaptic change over time [54]. Otherwise, we did not identify significant differences in SYN (SYN-1), GAP-43 and MAP-2 protein levels on each time point (*p* > 0.05). Similarly, the time-dependent synaptic change did not relate to each kind of synaptic junctions [55].

In this study, we assessed the synaptic ultrastructure of all groups on Day 14 post-cerebral ischemia using TEM. We found a clear and intact synaptic ultrastructure in the high magnification micrographs from sham group, and a disorganized synaptic ultrastructure in all the MCAO groups (Fig. 6). The synaptic disorganization was markedly ameliorated in the group that received the combination treatment of BYHWD and physical exercise.

## Conclusions

In summary, we conclude that BYHWD could be a potential therapeutic pharmaceutical approach, which can effectively facilitate neurorehabilitation following cerebral ischemia insult through improved synaptic plasticity.

## Additional files

Additional file 1: Chemical fingerprints of the BYHWD ingredients. UPLC-PAD/ESI-MS chromatogram of Buyang Huanwu decoction is shown in panel *a*: U-HPLC-PAD chromatography (*upper*) and ESI-MS (positive) total ion current (*bottom*). In other panels, UPLC-ESI-MS chromatogram of Buyang Huanwu decoction (*uppers* in panels *b*, *c*, *d*, *e*, *f*, *g*, *h*) and red peony root (*bottom* in panel *b*), Astragalus membranaceus (*bottom* in panel *c*), Chinese angelica root (*bottom* in panel *d*), Ligusticum wallichii (*bottom* in panel *e*), earthworm (*bottom* in panel *f*), safflower (*bottom* in panel *g*), peach seed (*bottom* in panel *h*) are displayed. (TIFF 8833 kb)

Additional file 2: Modified neurobehavioral assessment score. (PDF 15 kb)

## Abbreviations

BYHWD: Buyang Huanwu decoction
CBF: Cerebral blood flow
CCA: Common carotid artery
CNS: Central nervous system
ECA: External carotid artery
ICA: Internal carotid artery
LDF: Laser Doppler flowmetry
MCA: Middle cerebral artery
MCAO: Middle cerebral artery occlusion
TEM: Transmission electron microscope;
