# Supplementary figures and images for: Buyang Huanwu decoction facilitates neurorehabilitation through an improvement of synaptic plasticity in cerebral ischemic rats
Source: BMC Complement Altern Med. 2017 Mar 28;17:173. doi: 10.1186/s12906-017-1680-9 (PMC5371213; doi:10.1186/s12906-017-1680-9)

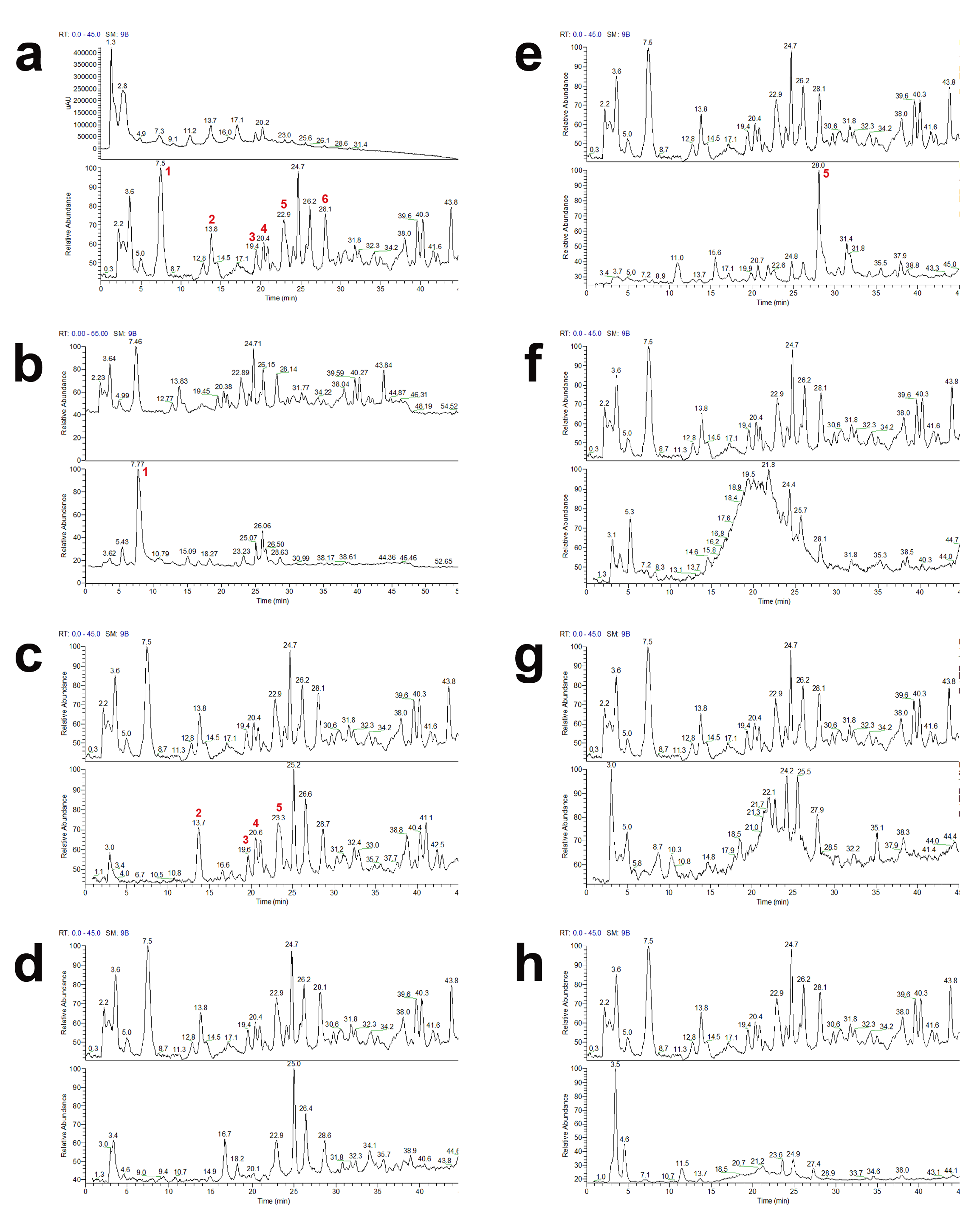

Supplement: Supplementary file 1 — Chemical fingerprints of the BYHWD ingredients. UPLC-PAD/ESI-MS chromatogram of Buyang Huanwu decoction is shown in panel a: U-HPLC-PAD chromatography (upper) and ESI-MS (positive) total ion current (bottom). In other panels, UPLC-ESI-MS chromatogram of Buyang Huanwu decoction (uppers in panels b, c, d, e, f, g, h) and red peony root (bottom in panel b), Astragalus membranaceus (bottom in panel c), Chinese angelica root (bottom in panel d), Ligusticum wallichii (bottom in panel e), earthworm (bottom in panel f), safflower (bottom in panel g), peach seed (bottom in panel h) are displayed. (TIFF 8833 kb) [file 12906_2017_1680_MOESM1_ESM.tif]
