# Supplementary material for: Buyang Huanwu decoction facilitates neurorehabilitation through an improvement of synaptic plasticity in cerebral ischemic rats
Source: BMC Complement Altern Med. 2017 Mar 28;17:173. doi: 10.1186/s12906-017-1680-9 (PMC5371213; doi:10.1186/s12906-017-1680-9)
Supplement: Supplementary file 2 — Modified neurobehavioral assessment score. (PDF 15 kb) [file 12906_2017_1680_MOESM2_ESM.pdf]

Modified neurobehavioral assessment score

Function	Score
**Motor**	
Raising rat via tail:	
● Flexion (forelimb)	1
● Flexion (hindlimb)	1
● Head movement within 30s	1
**Walk**	
● Normal walk	0
● Inability to walk straight	1
● Circling walk	2
● Inability to walk, and, fall down to one side	3
**Sensory**	
● Placing reaction (visual and tactile test)	1
● Proprioceptive sensation	1

**Beam balance**	
● Steady posture	0
● Grasping beam	1
● Hugging beam (with one limb falling down)	2
● Two limbs falling down or spinning on beam (within 60 s)	3
● Attempting to balance but falling of (within 40 s)	4
● Attempting to balance but falling off (within 20 s)	5
● Falling off (within 20 s)	6
**Absence of reflex and abnormal movements**	
● Pinna reflex	1
● Corneal reflex	1
● Startle reflex (through a brief noise from snapping a clipboard paper)	1
● Seizures, myoclonus, myodystony	1

Note: Maximum score: 18; mild neurological deficit: 1-6, moderate neurological deficit: 7-12, severe neurological deficit: 13-18.
